# Catastrophic Health Expenditure and Impoverishment from Non-Communicable Diseases: A comparison of Private and Public Health Facilities in Ekiti State, Southwest Nigeria

**DOI:** 10.4314/ejhs.v32i5.15

**Published:** 2022-09

**Authors:** Tope Michael Ipinnimo, Kabir Adekunle Durowade

**Affiliations:** 1 Department of Community Medicine, Federal Teaching Hospital, Ido-Ekiti, Ekiti State, Nigeria; 2 Department of Community Medicine, Afe Babalola University, Ado-Ekiti, Nigeria and Department of Community Medicine, Federal Teaching Hospital, Ido-Ekiti, Nigeria

**Keywords:** Catastrophic health expenditure, impoverishment, Nigeria, non-communicable diseases

## Abstract

**Background:**

Catastrophic health expenditure and impoverishment are the outcomes of poor financing mechanisms. Little is known about the prevalence and predictors of these outcomes among non-communicable disease patients in private and public health facilities.

**Methods:**

A health facility-based comparative cross-sectional study was conducted among 360 patients with non-communicable diseases (180 per group) selected through multistage sampling. Data were collected with a semi-structured, interviewer-administered questionnaire and analyzed with IBM SPSS for Windows, Version 22.0. Two prevalences of catastrophic health expenditure were calculated utilizing both the World Bank (CHE1) and the WHO (CHE2) methodological thresholds.

**Results:**

The prevalence of CHE1 (Private:42.2%, Public:21.7%, p<0.001) and CHE2 (Private:46.8%, Public:28.0%, p<0.001) were higher in private health facilities. However, there was no significant difference between the proportion of impoverishment (Private:24.3%, Public:30.9%, p=0.170). The identified predictors were occupation, number of complications and clinic visits for catastrophic health expenditure and socioeconomic status for impoverishment in private health facilities. Level of education, occupation, socioeconomic status, number of complications and alcohol predicted catastrophic health expenditure while the level of education, socioeconomic status and the number of admissions predicted impoverishment in public health facilities.

**Conclusions:**

Catastrophic health expenditure and impoverishment were high among the patients, with the former more prevalent in private health facilities. Therefore, we recommend expanding the coverage and scope of national health insurance among these patients to provide them with financial risk protection. Identified predictors should be taken into account by the government and other stakeholders when designing policies to limit catastrophic health expenditure and impoverishment among them.

## Introduction

Globally, non-communicable diseases (NCDs) are the leading cause of death and it exacts huge healthcare costs on patients and their household, forcing millions of people into poverty with suppressing development (1). In Nigeria, the cost of illness is borne majorly by individuals as the method of healthcare financing is still mostly out-of-pocket (OOP) (2). Poverty rate is high with more than 50% of Nigerians living below the international poverty line, most of them with no health insurance cover (3). The coverage of the national health insurance scheme (NHIS) is very low, with 5% of the Nigerian population and about 27% of hypertensive patients covered, leaving majority of the population including the most vulnerable exposed to catastrophic health expenditure (CHE) and impoverishment as a result of healthcare spending (4–6).

Health expenditure is said to be catastrophic when it is ≥40% of the annual household income remaining after subsistence needs have been met (7). According to the World Bank, household health expenditure >10% of household income is also said to be catastrophic (8). The proportion of households that fall below the national poverty line as a result of health expenditure is another approach used to assess the economic burden of OOP, and this is known as the impoverishment (9). Impoverishment occurs when a patient who is not poor, crosses below the international poverty line after paying for healthcare service (10).

A study on catastrophic health spending in 133 countries showed that 808 million people incurred CHE making the estimated global incidence of CHE to be 11.7% (11). Also, 23% of households experienced CHE and 4% experience impoverishment in Uganda. In Kenya, medication management of NCDs (hypertension or diabetes) required about 1% to 2% and 8% to 10% of the average annual income in the public and private sectors respectively (12). In Nigeria, the prevalence of CHE from the isolated cost of managing diabetes and/or hypertension was 72.6% while 48.2% were impoverished (13). Similarly, only 21.9% of patients with chronic diseases in Ile-Ife, Nigeria spent less than 10% of their monthly income on health while over 40% of them spent more than 40% of it (14).

Furthermore, enrolment in health insurance schemes have been linked to a reduced likelihood of CHE (12,15,16). On the contrary, factors that increased the likelihood of CHE were in-patient service especially in private hospitals (15), living in a rural area (17), or slums (10,15), unemployment or manual worker (17,18), low level of education (15,17,19) and socioeconomic status (10,20), households with the main income earner older than 55 years (15), female household head (15,19), large household size (17,18), household with elderly (10,15,17,21), and household with children (15,19). Levels of education, socioeconomic status and employment (13) as well as living in the slum (10) were found to affect impoverishment. Both CHE and impoverishment are also sensitive to methodology and threshold used in calculation as well as definitions of key indicators (15,16).

Little is known on how these factors and the other determinants affect CHE and impoverishment among NCDs patients in private and public health facilities (HF) in developing countries, especially Nigeria. The findings from this study will guide the government, other public stakeholders and private collaborators in developing policies that would protect against the financial risk of OOP expenditure among patients with NCDs therefore, getting closer to achieving a target of the Sustainable Development Goals. The study will also help to enrich the literature in addition to identifying economic gap in the care of NCDs between private and public HF in Nigeria. This study aims to determine and compare the prevalence of CHE and impoverishment resulting from the cost of managing NCDs and identify associated factors among patients accessing care in private and public HF in Ekiti State, Nigeria.

## Materials and Methods

This study was carried out in private and public HF within the capital of Ekiti State. The State lies in the southwest region of Nigeria and has a population of 2,737,186 according to 2006 census (3,821,378 in 2019 projected population) (22). Ekiti State is one of the poorest in Nigeria, with a poverty rate of under $1.25/day of about 65% (23). Healthcare financing in Ekiti State is not different from what is seen in Nigeria, as the majority of the population pay for their healthcare OOP. The NHIS coverage is low and only limited to federal government workers and workers in large organizations (24). The majority of public HF operate drug revolving funds to improve access and quality of medications.

This study used a health facility-based comparative cross-sectional design and the inclusion criteria were patients with hypertension and/or diabetes ≥18years of age, who had been on treatment for their illness for at least three months. Patients who were pregnant or too ill to respond were excluded from the study.

One hundred and eighty was calculated as the minimum sample size for each group after using the formula for a comparative study and assuming a non-response rate of 10% (25). A total of 360 patients were selected using a multistage sampling technique. In the first stage, the public HF were selected using stratified sampling after they have been stratified into primary, secondary and tertiary HF. Ten out of a total of twenty-eight public primary HF were selected by balloting. No public secondary HF was selected as there was no functional HF in this category while the only public tertiary HF was selected. Private HF were similarly selected by stratified sampling. Twenty-three out of the sixty-four private HF providing primary healthcare were selected by balloting, fifteen out of the forty-two private hospitals providing secondary healthcare were selected by balloting and the only private HF providing tertiary care was selected.

The number of patients interviewed per HF in each arm was based on proportionate allocation using the average number of patients seen in a month. In the second stage, patients were selected using systematic sampling technique. The average clinic attendance in a month was used as the sampling frame. The first patient was selected using a simple random sampling technique by balloting, after which the subsequent patients were selected by adding sampling interval until the required sample size was obtained.

The study instrument used was a semi-structured, interviewer-administered questionnaire, the content was adapted from studies by Pavel *et al* (26) and the World Bank Living Standard Measurement Survey (LSMS) questionnaires (27,28). The questionnaire included questions on sociodemographic variables as well as clinical variables such as frequency of visit to the HF, history of smoking and exercise in addition to intake of alcohol, salt, fruits and vegetables. Heights were measured with a portable stadiometer and weights with a well-calibrated Omron HN289 digital scale to determine patients' body mass index (BMI). There were questions on total monthly income from all sources, ownership of some household assets and monthly expenditures on food and other products. Data on the direct cost of managing NCDs such as the costs of consultations, laboratory and radiological investigations, medications, consumables, transportation to clinics, hospitalization and other money paid directly for the care of NCDs were collected during the interviews with the patients. Data were collected between October and November 2019.

**Data management and analysis**: Data entry and analysis were done with IBM SPSS Statistics for Window, Version 22.0 (IBM Corp., Armonk, N.Y., USA). Sociodemographic, economic and clinical variables were presented using frequency tables and percentages. Summary statistics such as mean (standard deviation) and median (interquartile range) were used for continuous variables such as age and monthly income respectively. The patients socioeconomic status was determined through the wealth scores, based on the ownership of some asset using “principal component analysis”, patients were then divided into five quintiles based on their wealth scores at one end ‘the poorest’ and at the other “the richest” (10).

The OOP expenditure on NCDs was calculated by summing the direct cost components of managing NCDs as described above. Two prevalences of CHE as a result of cost of managing NCDs were calculated. CHE as a result of cost of managing NCDs 1 (CHE1 -according to World Bank threshold) was calculated by using a ratio of health expenditure on NCDs (>10%) to the total income, while CHE as a result of cost of managing NCDs 2 (CHE2 -according to WHO threshold) was calculated by using a ratio of health expenditure on NCDs (≥40%) to the non-food consumption expenditure.

The CHE1 was calculated with the formula:OOP_h_/Inc_t_

OOP_h_= Out-of-pocket health expenditure on NCDs

Inc_t_= Total income

CHE1 is present if OOP_h_/Inc_t_ ≥ 0.1 but absent if OOP_h_/Inc_t_ < 0.1(8)

The CHE2 was calculated with the formula: OOP_h_/(Exp_t_ - Exp_f_)

Exp_t_= Total household expenditure

Exp_f_= Household food expenditure

CHE2 is present if OOP_h_/(Exp_t_ - Exp_f_) ≥ 0.4 and absent if OOP_h_/(Exp_t_ - Exp_f_) < 0.4 (7). Sensitivity analysis of the threshold of CHE1&2 was done to improve the robustness of the study. CHE2 was used for further analysis because the WHO recommends the use of non-food expenditure as the measure of household's capacity to pay because food is seen as a basic necessity and composes a major share of household expenditure (29,30).

The proportion of patients pushed into poverty was generated after setting the absolute poverty line at $1.90 per head per day (₦684/day using ₦360 exchange rate). This is the World Bank international poverty line for 2018. A patient was said to be pushed into poverty when OOP expenditure on NCDs dropped the total income below the international poverty line of $1.90 per person per day (Inc_t_ – OOP_h_<$1.90 per head per day) (31). The percentage of individuals that fall below this poverty line after removing payments for care of NCDs is the proportion pushed into poverty.

The distribution of extent of CHE and impoverishment among NCDs patients in private and public HF and across patients' variables were compared using the Chi-square test. Binary logistic regression was used to study the predictors of CHE and impoverishment and a P-value of ≤0.05 was taken as statistical significance.

**Ethical consideration**: Institutional ethical approval for the study was obtained from the Ethics and Research Review Committee of the Federal Teaching Hospital, Ido-Ekiti. Written consent was obtained from each selected participant.

## Results

A total of 360 patients were interviewed but 348 (96.7%) responded with complete data (173 in private and 175 in public HF). The sociodemographic characteristics of respondent presented in table 1 shows that the mean age of the patients was 59.6±10.8years (Private: 59.5±10.5years, Public: 59.7±11.2 years, p=0.910). There was a statistically significant difference in the level of education (p=0.003), income (p=0.003), OOP expenditure (p<0.001) and number of days of exercise (p<0.001) among the participants in the two groups. Other sociodemographic and clinical variables did not show any significant difference (p>0.05).

**Table 1:** Socio-demographic, economic and clinical characteristics of the participants

Variables	Health Facility			Test	p-value

	Private (%) n= 173	Public (%) n= 175	Total (%) N=348		
***Mean age±SD(Years)***	*59.51±10.49*	*59.65±11.21*	*59.58±10.84*	*-0.113* *	*0.910*
**Sex**					
Male	78 (45.1)	79 (45.1)	157 (45.1)	<0.001^X^	0.992
Female	95 (54.9)	96 (54.9)	191 (54.9)		
**Level of Education**					
No formal education	7 (4.0)	20 (11.4)	27 (7.8)	13.807^X^	**0.003**
Primary education	27 (15.6)	38 (21.7)	65 (18.7)		
Secondary education	52 (30.1)	58 (33.2)	110 (31.5)		
Tertiary education	87 (50.3)	59 (33.7)	146 (42.0)		
**Occupation**					
Formal	59 (34.1)	48 (27.4)	107 (30.7)	2.767^X^	0.251
Informal	76 (43.9)	92 (52.6)	168 (48.3)		
Retired/Unemployed	38 (22.0)	35 (20.0)	73 (21.0)		
***Median income (IQR)* (₦)**	*42,000 (40,000)*	*35,000 (40,200)*	*40,000 (41,750)*	12326.00^M^	**0.003**
**Socioeconomic Status**					
Poorest	29 (16.8)	40 (22.9)	69 (19.8)	2.728^X^	0.604
Poor	35 (20.2)	35 (20.0)	70 (20.2)		
Average	34 (19.7)	35 (20.0)	69 (19.8)		
Rich	36 (20.8)	34 (19.4)	70 (20.1)		
Richest	39 (22.5)	31 (17.7)	70 (20.1)		
**Payment Method**					
NHIS	17 (9.8)	10 (5.7)	27 (7.8)	2.056^X^	0.152
OOP	156 (90.2)	165 (94.3)	321 (92.2)		
**Number of Complications**					
0	117 (67.6)	114 (65.1)	231 (66.4)	0.537^X^	0.764
1	34 (19.7)	40 (22.9)	74 (21.2)		
2 or more	22 (12.7)	21 (12.0)	43 (12.4)		
**Exercise**					
<3 days per week	161 (93.1)	134 (76.6)	295 (84.8)	18.328^X^	**<0.001**
≥3 days per week	12 (6.9)	41 (23.4)	53 (15.2)		
**Alcohol**					
<14 units per week	152 (87.9)	151 (86.3)	303 (87.1)	0.192^X^	0.661
≥14 units per week	21 (12.1)	24 (13.7)	45 (12.9)		
**Smoking**					
Not smoking	157 (90.8)	159 (90.9)	316 (90.8)	0.001^X^	0.973
Smoking	16 (9.2)	16 (9.1)	32 (9.2)		
**Body Mass Index**					
Normal	50 (28.9)	62 (35.4)	112 (32.2)	1.760^X^	0.415
Overweight	93 (53.8)	87 (49.7)	180 (51.7)		
Obese	30 (17.3)	26 (14.9)	56 (16.1)		
**NOCV (in 1 month)**					
0	5 (2.9)	5 (2.9)	10 (2.9)	5.830^X^	0.054
1	134 (77.4)	116 (66.2)	250 (71.8)		
2 or more	34 (19.7)	54 (30.9)	88 (25.3)		
**NOA (in 1 month)**					
0	155 (89.6)	158 (90.3)	313 (89.9)	0.046^X^	0.830
1	18 (10.4)	17 (9.7)	35 (10.1)		
**Median*(IQR)* OOP payment (₦)**	11,750.00	6,200.00	8,200.00	7832.00^M^	**<0.001**
	(6,750.00)	(5,016.00)	(7,475.00)		

X Chi-square test

* T-test

M Mann-Whitney U test

SD--Standard deviation, IQR--Interquartile Range, NOCV--Number of Clinic Visits, NOA--Number of Admissions, OOP--Out-of-pocket

In Table 2, the prevalence of CHE1 and CHE2 were higher in private HF than in public HF (Private:42.2%, Public:21.7%, p<0.001) and (Private:46.8%, Public:28.0%, p<0.001) respectively. Results of sensitivity analysis of CHE1 and CHE2 continue to show a significantly higher prevalence of CHE in private HF than in public HF except for CHE2 analysis at 50% threshold (p=0.084). The proportion of impoverished patients as a result of cost of managing NCDs was lower in private HF (Private:24.3%, Public:30.9%) but this was not statistically significant (p=0.170).

**Table 2:** Prevalence of catastrophic health expenditure (CHEN) using Sensitive Analysis and Impoverishment as a result of the cost of care of NCDs among the participants

Variable	Health Facility			*X* ^2^	p-value

	Private (%) n= 173	Public (%) n= 175	Total (%) N=348		
**CHEN1**					
**5%** [YES]	98 (56.6)	52 (29.7)	150 (43.1)	25.732	**<0.001**
[NO]	75 (43.4)	123 (70.3)	198 (56.9)		

*10% (YES)	73 (42.2)	38 (21.7)	111 (31.9)	16.801	**<0.001**
(NO)	100 (57.8)	137 (78.3)	237 (68.1)		

**15%** (YES)	54 (31.2)	32 (18.3)	86 (24.7)	7.815	**0.005**
(N0)	119 (68.8)	143 (81.7)	262 (75.3)		
**CHEN2**					
**30%** (YES)	101 (58.4)	62 (35.4)	163 (46.8)	18.407	**<0.001**
(NO)	72 (41.6)	113 (64.6)	185 (53.2)		

**40% (YES)	81 (46.8)	49 (28.0)	130 (37.4)	13.169	**<0.001**
(NO)	92 (53.2)	126 (72.0)	218 (62.6)		

**50%** (YES)	57 (32.9)	43 (24.6)	100 (28.7)	2.981	0.084
(NO)	116 (67.1)	132 (75.4)	248 (71.3)		
**Impoverished**					
[YES]	42 (24.3)	54 (30.9)	96 (27.6)	1.885	0.170
[NO]	131 (75.7)	121 (69.1)	252 (72.4)		

X^2^--Chi-square test

* threshold according to World Bank^58^

** threshold according to WHO^88^

Tables 3 and 4 show the sociodemographic, economic and clinical factors associated with catastrophic health expenditure (CHE2) and the risk of being impoverished at bivariate level of analysis. Details of these factors are presented in these tables.

**Table 3:** Patients' characteristics associated with catastrophic health expenditure (CHEN2)

Variable	Health Facility							

	Private (n=173)				Public (n=175)			
	CHEN2		X^2^	p-value	CHEN2		X^2^	p-value
	
	YES (*n*=81)	NO (*n*=92)			YES (*n*=49)	NO (*n*=126)		
**Sex**								
Male	28 (35.9)	50 (64.1)	6.807	**0.009**	20 (25.3)	59 (74.7)	0.514	0.473
Female	53 (55.8)	42 (44.2)			29 (30.2)	67 (69.8)		
**Level of Education**								
No formal education	6 (85.7)	1 (14.3)	55.688^f^	**<0.001**	15 (71.4)	6 (28.6)	33.619	**<0.001**
Primary education	24 (88.9)	3 (11.1)			15 (39.5)	23 (60.5)		
Secondary education	33 (63.5)	19 (36.5)			14 (24.6)	43 (75.4)		
Tertiary education	18 (20.7)	69 (79.3)			5 (8.5)	54 (91.5)		
**Marital Status**								
Unmarried	29 (55.8)	23 (44.2)	2.391	0.122	21 (42.0)	29 (58.0)	6.806	**0.009**
Married	52 (43.0)	69 (57.0)			28 (22.4)	97 (77.6)		
**Occupation**								
Formal	2 (3.4)	57 (96.6)	78.843^f^	**<0.001**	3 (6.2)	45 (93.8)	18.228^f^	**<0.001**
Informal	53 (69.7)	23 (30.3)			32 (34.8)	60 (65.2)		
Retired/Unemployed	26 (68.4)	12 (31.6)			14 (40.0)	21 (60.0)		
**Socioeconomic Status**								
Poorest	27 (93.1)	2 (6.9)	67.484^f^	**<0.001**	25 (62.5)	15 (37.5)	35.873^f^	**<0.001**
Poor	20 (57.1)	15 (42.9)			10 (28.6)	25 (71.4)		
Average	17 (50.0)	17 (50.0)			9 (25.7)	26 (74.3)		
Rich	16 (44.4)	20 (55.6)			3 (8.8)	31 (91.2)		
Richest	1 (2.6)	38 (97.4)			2 (6.5)	29 (93.5)		
**Payment Method**								
NHIS	0 (0.0)	17 (100.0)	14.579^Y^	**<0.001**	1 (10.0)	9 (90.0)	0.889^Y^	0.346
OOP	81 (51.9)	75 (48.1)			48 (29.1)	117 (70.9)		
**NOC**								
0	45 (38.5)	72 (61.5)	21.747^f^	**<0.001**	22 (19.3)	92 (80.7)	20.258	**<0.001**
1	16 (47.1)	18 (52.9)			13 (32.5)	27 (67.5)		
2 or more	20 (90.9)	2 (9.1)			14 (66.7)	7 (33.3)		
**Exercise**								
<3 days per week	74 (46.0)	87 (54.0)	0.686	0.407	41 (30.6)	93 (69.4)	1.913	0.167
≥3 days per week	7 (58.3)	5 (41.7)			8 (19.5)	33 (80.5)		
**Alcohol**								
<14 units per week	69 (45.4)	83 (54.6)	1.023	0.312	35 (23.2)	116 (76.8)	12.695	**<0.001**
≥14 units per week	12 (57.1)	9 (42.9)			14 (58.3)	10 (41.7)		
**Smoking**								
Not smoking	72 (45.9)	85 (54.1)	0.630	0.428	39 (24.5)	120 (75.5)	10.397	**0.001**
Smoking	9 (56.2)	7 (43.8)			10 (62.5)	6 (37.5)		
**Body Mass Index**								
Normal	22 (44.0)	28 (56.0)	1.431	0.489	13 (21.0)	49 (79.0)	5.779	0.056
Overweight	42 (45.2)	51 (54.8)			24 (27.6)	63 (72.4)		
Obese	17 (56.7)	13 (43.3)			12 (46.2)	14 (53.8)		
**NOCV (in 1 month)**							
0	1 (20.0)	4 (80.0)	12.859^f^	**0.001**	2 (40.0)	3 (60.0)	26.188^f^	**<0.001**
1	55 (41.0)	79 (59.0)			18 (15.5)	98 (84.5)		
2 or more	25 (73.5)	9 (26.5)			29 (53.7)	25 (46.3)		
**NOA (in 1 month)**								
0	65 (41.9)	90 (58.1)	12.456^Y^	**<0.001**	32 (20.3)	126 (79.7)	44.543^Y^	**<0.001**
1	16 (88.9)	2 (11.1)			17(100.0)	0 (0.0)		

X^2^--Chi-square test

Y Continuity correction

f Fisher's exact test

NOC--Number of Complications, NOCV--Number of Clinic Visits, NOA--Number of Admissions

**Table 4:** Patients' characteristics associated with impoverishment

Variable	Health Facility							

	Private (n=173)				Public (n=175)			

	Impoverished		X^2^	p-value	Impoverished		X^2^	p-value
	
	YES (*n*=42)	NO (*n*=131)			YES (*n*=54)	NO (*n*=121)		
**Sex**								
Male	13 (16.7)	65 (83.3)	4.476	**0.034**	25 (31.6)	54 (68.4)	0.042	0.838
Female	29 (30.5)	66 (69.5)			29 (30.2)	67 (69.8)		
**Level of Education**								
No formal education	6 (85.7)	1 (14.3)	46.849^f^	**<0.001**	18 (85.7)	3 (14.3)	56.819^f^	**<0.001**
Primary education	13 (48.1)	14 (51.9)			20 (52.6)	18 (47.4)		
Secondary education	19 (36.5)	33 (63.5)			12 (21.1)	45 (78.9)		
Tertiary education	4 (4.6)	83 (95.4)			4 (6.8)	55 (93.2)		
**Marital Status**								
Unmarried	15 (28.8)	37 (71.2)	0.844	0.358	25 (50.0)	25 (50.0)	12.023	**0.001**
Married	27 (22.3)	94 (77.7)			29 (23.2)	96 (76.8)		
**Occupation**								
Formal	0 (0.0)	59 (100.0)	37.991^f^	**<0.001**	3 (6.2)	45 (93.8)	21.908^f^	**<0.001**
Informal	28 (36.8)	48 (63.2)			36 (39.1)	56 (60.9)		
Retired/Unemployed	14 (36.8)	24 (63.2)			15 (42.9)	20 (57.1)		
**Socioeconomic Status**								
Poorest	24 (82.8)	5 (17.2)	64.503^f^	**<0.001**	32 (80.0)	8 (20.0)	79.298^f^	**<0.001**
Poor	5 (14.3)	30 (85.7)			14 (40.0)	21 (60.0)		
Average	6 (17.6)	28 (82.4)			7 (20.0)	28 (80.0)		
Rich	7 (19.4)	29 (80.6)			0 (0.0)	34 (100.0)		
Richest	0 (0.0)	39 (100.0)			1(3.2)	30 (96.8)		
**Payment Method**								
NHIS	0 (0.0)	17 (100.0)	4.669^Y^	**0.031**	1 (10.0)	9 (90.0)	1.250^Y^	0.264
OOP	42 (26.9)	114 (73.1)			53 (32.1)	112 (67.9)		
**NOC**								
0	24 (20.5)	93 (79.5)	3.138	0.208	30 (26.3)	84 (73.7)	4.189	0.123
1	10 (29.4)	24 (70.6)			14 (35.0)	26 (65.0)		
2 or more	8 (36.4)	14 (63.6)			10 (47.6)	11 (52.4)		
**Exercise**								
<3 days per week	37 (23.0)	124 (77.0)	2.121	0.145	42 (31.3)	92 (68.7)	0.063	0.801
≥3 days per week	5 (41.7)	7 (58.3)			12 (29.3)	29 (70.7)		
**Alcohol**								
<14 units per week	36 (23.7)	116 (76.3)	0.240	0.624	41 (27.2)	110 (72.8)	7.083	**0.008**
≥14 units per week	6 (28.6)	15 (71.4)			13 (54.2)	11 (45.8)		
**Smoking**								
Not smoking	36 (22.9)	121 (77.1)	1.677	0.195	46 (28.9)	113 (71.1)	3.025	0.082
Smoking	6 (37.5)	10 (62.5)			8 (50.0)	8 (50.0)		
**Body Mass Index**								
Normal	8 (16.0)	42 (84.0)	2.741	0.254	14 (22.6)	48 (77.4)	6.458	**0.040**
Overweight	25 (26.9)	68 (73.1)			27 (31.0)	60 (69.0)		
Obese	9 (30.0)	21 (70.0)			13 (50.0)	13 (50.0)		
**NOCV (in 1 month)**								
0	1 (20.0)	4 (80.0)	0.755^f^	0.799	1 (20.0)	4 (80.0)	10.968	**0.004**
1	31 (23.1)	103 (76.9)			27 (23.3)	89 (76.7)		
2 or more	10 (29.4)	24 (70.6)			26 (48.1)	28 (51.9)		
**NOA (in 1 month)**								
0	29 (18.7)	126 (81.3)	25.121	**<0.001**	40 (25.3)	118 (74.7)	20.806	**<0.001**
1	13 (72.2)	5 (27.8)			14 (82.4)	3 (17.6)		

X^2^--Chi-square test

Y Continuity correction

f Fisher's exact test

NOC--Number of Complications, NOCV--Number of Clinic Visits, NOA--Number of Admissions

Table 5 shows the result of multivariate logistic regression analysis indicating the predictors of CHE. In private HF, patients with 2 or more complications were more likely to experience CHE from cost of managing NCDs than those without complications (OR=11.911; 95%CI=1.364–104.033). Patients employed in formal occupation than those that were unemployed/retired (OR=0.009; 95%CI=0.001–0.299) and those who attended one clinic than those who attended two or more clinics in the last one month (OR=0.033; 95%CI=0.001–0.869) were less likely to experience CHE from cost of managing NCDs.

**Table 5:** Logistic regression relating catastrophic health expenditure to predictor variables

Variable	Health Facility							

	Private (n= 173)				Public (n= 175)			
	Odd Ratio	p-value	95%CI		Odd Ratio	p-value	95%CI	
	
			Lower	Higher			Lower	Higher
Sex								
Male R	1.000							
Female	10.525	0.058	0.921	120.264	-	-	-	-
**Level of Education**								
No formal education R	1.000				1.000			
Primary education	8.947	0.999	<0.001	14.739	0.294	0.253	0.294	2.399
Secondary education	2.327	0.999	<0.001	14.739	0.045	**0.031**	0.003	0.758
Tertiary education	0.527	0.999	<0.001	14.739	0.015	**0.039**	<0.001	0.811
**Marital Status**								
Unmarried	-	-	-	-	0.400	0.376	0.053	3.039
Married R					1.000			
**Occupation**								
Formal	0.009	**0.008**	0.001	0.299	0.098	0.110	0.006	1.693
Informal	0.454	0.448	0.059	3.497	0.065	**0.021**	0.006	0.662
Retired/Unemployed R	1.000				1.000			
**Socioeconomic Status**								
Poorest R	1.000				1.000			
Poor	<0.001	0.998	<0.001	20.232	0.079	**0.035**	0.008	0.836
Average	<0.001	0.998	<0.001	19.498	0.520	0.605	0.043	6.229
Rich	<0.001	0.998	<0.001	22.583	0.001	0.097	<0.001	3.950
Richest	<0.001	0.996	<0.001	39.632	0.105	0.233	0.003	4.263
**Payment Method**								
NHIS	<0.001	0.998	0.001	20.658	-	-	-	-
OOP R	1.000							
**Number of Complications**								
0 R	1.000				1.000			
1	2.736	0.453	0.198	37.838	0.717	0.791	0.062	8.354
2 or more	11.911	**0.025**	1.364	104.033	28.164	**0.014**	1.958	405.063
**Alcohol**								
<14 units per week	-	-	-	-	0.004	**0.012**	<0.001	0.293
≥14 units per week R					1.000			
**Smoking**								
Not smoking	-	-	-	-	0.001	0.112	<0.001	5.518
Smoking R					1.000			
**NOCV (in 1 month)**								
0	<0.001	0.997	<0.001	26.083	4.559	0.323	0.225	92.557
1	0.033	**0.041**	0.001	0.869	0.922	0.945	0.092	9.272
2 or more R	1.000				1.000			
**NOA (in 1 month)**								
0	<0.001	0.997	<0.001	18.320	<0.001	0.997	<0.001	28.053
1 R	1.000				1.000			

95% CI --95% Confidence Interval

R Reference Variable

- --Excluded Variable, NOCV--Number of Clinic Visits, NOA--Number of Admissions

In public HF, patients with 2 or more complications were more likely to experience CHE from cost of managing NCDs than those without complications (OR=28.164; 95%CI=1.958–405.063). Patients with secondary and tertiary education than those with no formal education (Secondary: OR=0.045; 95%CI=0.003–0.758, Tertiary: OR=0.015; 95%CI=<0.001–0.811), patients drinking <14 units of alcohol per week than those drinking ≥14 units per week (OR=0.004; 95%CI=<0.001–0.293), those employed in informal occupations than those who were unemployed/retired (OR=0.065; 95%CI=0.006–0.662) and patients with poor socioeconomic status than those from the poorest socioeconomic status (OR=0.079; 95%CI=0.008–0.836) were less likely to experience CHE from cost of managing NCDs.

Table 6 shows that in private HF, patients with poor and average socioeconomic status were 95.3% and 94.6% less likely to experience impoverishment than those in the poorest socioeconomic status (Poor: OR=0.047; 95%CI=0.008–0.295, Average: OR=0.054; 95%CI=0.008–0.370). In public HF, patients with secondary education than those with no formal education (OR=0.074; 95%CI=0.006–0.913), patients with poor (OR=0.247; 95%CI=0.058–1.047), average (OR=0.036; 95%CI=0.004–0.341) and richest socioeconomic status (OR=0.001; 95%CI=<0.001–0.361) than those from the poorest socioeconomic status, patients with no admission than those with admission (OR=0.014; 95%CI=<0.001–0.466) were less likely to experience impoverishment from cost of managing NCDs.

**Table 6:** Logistic regression relating impoverishment to predictor variables

Variable	Health Facility							

	Private (n= 173)				Public (n= 175)			

	Odd Ratio	p-value	95%CI		Odd Ratio	p-value	95%CI	
	
			Lower	Higher			Lower	Higher
**Sex**								
Male R	1.000							
Female	2.493	0.252	0.522	11.904	-	-	-	-
**Level of Education**								
No formal education R	1.000				1.000			
Primary education	<0.001	0.998	<0.001	18.410	0.294	0.325	0.026	3.377
Secondary education	<0.001	0.998	<0.001	18.905	0.074	**0.042**	0.006	0.913
Tertiary education	<0.001	0.998	<0.001	20.124	0.363	0.509	0.018	7.382
**Marital Status**								
Unmarried	-	-	-	-	2.273	0.259	0.547	9.453
Married R					1.000			
**Occupation**								
Formal	<0.001	0.997	<0.001	17.070	0.202	0.338	0.008	5.341
Informal	0.540	0.487	0.095	3.076	0.610	0.578	0.107	3.470
Retired/Unemployed R	1.000				1.0000			
**Socioeconomic Status**								
Poorest R	1.000				1.000			
Poor	0.047	**0.001**	0.008	0.295	0.247	**0.058**	0.058	1.047
Average	0.054	**0.003**	0.008	0.370	0.036	**0.004**	0.004	0.341
Rich	<0.001	0.996	<0.001	20.540	<0.001	0.997	<0.001	23.301
Richest	<0.001	0.995	<0.001	38.022	0.001	**0.020**	<0.001	0.361
**Payment Method**								
NHIS	<0.001	0.998	<0.001	16.391	-	-	-	-
OOP R	1.000							
**Alcohol**								
<14 units per week R					1.000			
≥14 units per week	-	-	-	-	4.217	0.280	0.309	57.477
**Body Mass Index**								
Normal	-	-	-	-	0.230	0.143	0.032	1.642
Overweight	-	-	-	-	0.220	0.121	0.032	1.490
Obese R					1.000			
**NOCV (in 1 month)**								
0	-	-	-	-	0.706	0.861	0.014	34.585
1	-	-	-	-	1.401	0.746	0.182	10.764
2 or more R					1.000			
**NOA (in 1 month)**								
0	<0.001	0.996	<0.001	21.512	0.014	**0.017**	<0.001	0.466
1 R	1.000				1.000			

95% CI --95% Confidence Interval

R Reference Variable

- --Excluded Variable, NOCV--Number of Clinic Visits, NOA--Number of Admissions

## Discussion

This study found the prevalence of CHE from NCDs to be higher in private than in public HF and this is similar to results from a previous study (32). This finding may be due to drug revolving funds operated by the public HF which must have improved access to medications that constitute a huge proportion of NCDs care expenditure (13,33). Additionally, the prevalence of CHE2 was higher than CHE1, this further confirms that the prevalence of CHE is sensitive to the method and threshold used (15). It also suggested that a large percentage of the patients' income were going into buying food items. Prevalence of CHE similar to the finding in this study has been reported in Nigeria (34). Other studies within and outside Nigeria have reported lower prevalence (10,16,35). The high prevalence of CHE seen in this study may be due to the high OOP spending among these patients. It was documented that CHE is low in countries where OOP is below 20% of the total health expenditures (36).

The proportion of patients in public HF (30.9%) who were impoverished was higher than in private HF (24.3%). This finding is higher than what was reported in Uganda (4%) and Vietnam (2-5%) (10,37). However, it is lower than what was found among patients with hypertension and/or diabetes in Nigeria (13). The higher prevalence of impoverishment in public HF than in private HF, although not statistically significant, may result from public HF patients earning significantly lesser income than private HF patients. These patients earn very close to the poverty line such that, a little financial stress will tilt them into poverty. Therefore, it would be appropriate to consider and protect poor patients against the effects of OOP expenditure such as CHE and impoverishment.

In this study, patients with a lower level of education and socioeconomic status were more likely to experience CHE and impoverishment. Likewise, NCDs patients who were not employed in the formal occupations were more likely to experience CHE. This is consistent with findings from previous studies that identified lower socioeconomic class (10,20), lower level of education (15,17,19) and being unemployed or a manual worker (17,18) as factors that increase the likelihood of CHE. Another study among patients with NCDs in Nigeria found these factors as strong predictors of impoverishment (13). Occupation and level of education are both indicators of socioeconomic status (38). Higher level of education will give individuals access to the right information that would improve their health, and higher socioeconomic status would give them the power to acquire wealth, which invariably will enable them to pay for the cost of managing their conditions without so much impact on other expenditures (13).

Furthermore, patients working in formal occupations may be protected from CHE because, the health insurance in Nigeria covers mainly these people, leaving out the vulnerable groups (24). In this study, NHIS was associated with a lower proportion of patients experiencing CHE and impoverishment in private HF, although this was not significant after logistic regression analysis. Previous studies have shown an inverse relationship between health insurance and CHE (15,16). However, similar association between NHIS and CHE or impoverishment was not found in public HF. A good proportion of patients registered under this scheme in public HF were still being pushed into CHE and impoverishment. A finding that is in line with a study conducted in Vietnam (10). It may be important to look into the scheme and its benefits package as it affects the public HF because it appears that the current package may not be offering full protection to enrollees with NCDs (13).

As regards the clinical variables, a higher number of complications, presence of hospital admission and higher number of clinic visits were identified predictors of CHE and impoverishment. This finding is similar to that of previous studies (15,21,39). Also, alcohol use >14 units per week increases the risk of experiencing CHE and impoverishment. No study was found that assessed alcohol use or other modifiable risk factor as a determinant of CHE and impoverishment. Modifiable risk factors could raise the likelihood of experiencing CHE and impoverishment by increasing the risk of developing complications and morbidities (38). NCDs patient with complication(s) would require more follow-up and in severe cases may need to be hospitalized. Studies have shown that hospitalization leads to CHE and impoverishment (21,39). Frequent clinic visits and hospital admission would bring about increased utilization of healthcare resources which is mainly settled by OOP payment in Nigeria (13).

The limitation of this study is that it relied upon self-reported information by patients which may be prone to bias. Although this was mitigated by limiting the recall period to a month and by verifying information from payment receipts and HF records.

There is a high prevalence of CHE and impoverishment among patients with NCDs, although CHE was more prevalent in private HF, impoverishment did not show any significant difference. The identified predictors were occupation, number of complications and clinic visits for CHE and socioeconomic status for impoverishment in private HF. In public HF, level of education, occupation, socioeconomic status, number of complications and alcohol use were predictive of CHE while the level of education, socioeconomic status and number of admissions were predictors of impoverishment. Therefore, it is important to expand the scope and coverage of NHIS among NCD patients to give full protection against CHE and impoverishment. Also, to reduce the burden of OOP expenditure from NCDs, patients should be encouraged to practice lifestyle modifications such as consumption of a safe level of alcohol. Lastly, other identified predictors should be taken into account by the government, public and private health facilities managers and other stakeholders when designing policies that would provide financial risk protection against OOP expenditure among patients with NCDs.
